# Nrh L11R single nucleotide polymorphism, a new prediction biomarker in breast cancer, impacts endoplasmic reticulum-dependent Ca^2+^ traffic and response to neoadjuvant chemotherapy

**DOI:** 10.1038/s41419-023-05917-7

**Published:** 2023-07-01

**Authors:** Minh Quang Duong, Rudy Gadet, Isabelle Treilleux, Stéphane Borel, Adrien Nougarède, Olivier Marcillat, Philippe Gonzalo, Ivan Mikaelian, Nikolay Popgeorgiev, Ruth Rimokh, Germain Gillet

**Affiliations:** 1 Université de Lyon, Université Claude Bernard Lyon 1, INSERM 1052, CNRS 5286, Centre Léon Bérard, Centre de Recherche en Cancérologie de Lyon, Lyon, 69008 France; 2 Département de Biopathologie, Centre Léon Bérard, Lyon, France; 3 Laboratoire de Biochimie, CHU de Saint-Etienne, Université de Lyon, Lyon, France; 4 Hospices civils de Lyon, Centre de Biologie Sud, Centre Hospitalier Lyon Sud, chemin du Grand Revoyet, 69495 Pierre Bénite, France; 5 Present Address: Division for Biology and Healthcare Technologies, CEA-LETI, MINATEC Campus, F-38054 Grenoble, France

**Keywords:** Apoptosis, Breast cancer

## Abstract

Overexpression of Bcl-2 proteins such as Bcl2L10, also referred to as Nrh, is associated with resistance to therapy and poor survival in various cancers, including breast cancer, lung cancer, and leukemia. The single nucleotide polymorphism (SNP) of BCL2L10 in its BH4 domain at position 11 (BCL2L10 Leu11Arg, rs2231292), corresponding to position 11 in the Nrh open reading frame, is reported to lower resistance towards chemotherapy, with patients showing better survival in the context of acute leukemia and colorectal cancer. Using cellular models and clinical data, we aimed to extend this knowledge to breast cancer. We report that the homozygous status of the Nrh Leu11Arg isoform (Nrh-R) is found in 9.7–11% percent of the clinical datasets studied. Furthermore, Nrh-R confers higher sensitivity towards Thapsigargin-induced cell death compared to the Nrh-L isoform, due to altered interactions with IP_3_R1 Ca^2+^ channels in the former case. Collectively, our data show that cells expressing the Nrh-R isoform are more prone to death triggered by Ca^2+^ stress inducers, compared to Nrh-L expressing cells. Analysis of breast cancer cohorts revealed that patients genotyped as Nrh-R/Nrh-R may have a better outcome. Overall, this study supports the notion that the rs2231292 Nrh SNP could be used as a predictive tool regarding chemoresistance, improving therapeutic decision-making processes. Moreover, it sheds new light on the contribution of the BH4 domain to the anti-apoptotic function of Nrh and identifies the IP_3_R1/Nrh complex as a potential therapeutic target in the context of breast cancer.

## Introduction

Since the discovery of Bcl-2 in the early 1980s, the role of Bcl-2 family proteins in cell death has been extensively studied [1]. Indeed, to cope with oncogenic stress, cancer cells often rely on the upregulation of anti-apoptotic Bcl-2 proteins. Anti-apoptotic Bcl-2 homologs have four homology domains referred to as Bcl-2 homology (BH) domains [2]. BH3, BH1, and BH2 domains are clustered at the level of an hydrophobic binding pocket that meditates most protein-protein interactions (PPi) involved in apoptosis control [3]. The BH4 domain appears to act as a guardian of the pocket accessibility [4] and was reported to interact with a number of partners outside the Bcl-2 family, contributing in this way to non-canonical functions, beyond apoptosis, such as Ca^2+^ homeostasis maintenance [5–7]. In this respect, the BH4 domain of both Bcl-2 and Bcl-2l10/Nrh was shown to interact with IP3R Ca^2+^ channels at the level of the endoplasmic reticulum (ER), which may indirectly dampen the unfolded protein response in a Ca^2+^-dependent manner [8, 9]. Furthermore, there is growing evidence that the BH4 domain is involved in resistance to chemotherapy [4, 9].

Bcl2L10, also referred to as Bcl-B or Nrh, was identified as an anti-apoptotic protein, upregulated in various cancers, including leukemia and breast cancer [8, 10–14]. In addition, Nrh expression was correlated with poor prognosis in several instances [8, 15]. However, Nrh was also reported to exert pro-apoptotic activity in some instances, although the underlying mechanism remains unclear [16, 17]. Interestingly, a single nucleotide polymorphism (SNP ^#^rs2231292) was described in the *BCL2L10* locus, at position 21 BCL2L10-L11 > R (position 11 with respect to Nrh ORF). This SNP is located at the level of the BH4-encoding region and has been associated with a higher risk of relapse and increased resistance to therapy in acute leukemia and colorectal cancer, respectively [18, 19]. However, the underlying mechanisms remain unknown.

We previously showed that Nrh expression is an independent marker of poor prognosis in breast cancer, being associated with shorter distant metastasis-free survival (DMFS). We also demonstrated that anti-apoptotic activity of Nrh was dependent on its BH4 domain [8]. This domain encompasses the rs2231292 polymorphism, giving rise to the Nrh-L or -R variant. Here, we sought to assess the significance of this polymorphism in the context of breast cancer. Analyses performed on patient cohorts indicate that although this polymorphism cannot be used as a predictor of outcome, the homozygous R/R character was prognostic of a better response to chemotherapy. Mechanistically, we show that the Nrh-R isoform, in contrast to the Nrh-L isoform, lacked the ability to associate with the N-terminal region of the IP3R1 Ca^2+^ channel and thus block the response to Ca^2+^ stress inducers. Collectively, our data provide a molecular explanation regarding current clinical data and identify the Nrh/IP3R1 complex as a prime therapeutic target in breast cancer.

## Results

### Nrh-L and Nrh-R differentially regulate cell death induced by calcium stress inducers

**Fig. 1:**
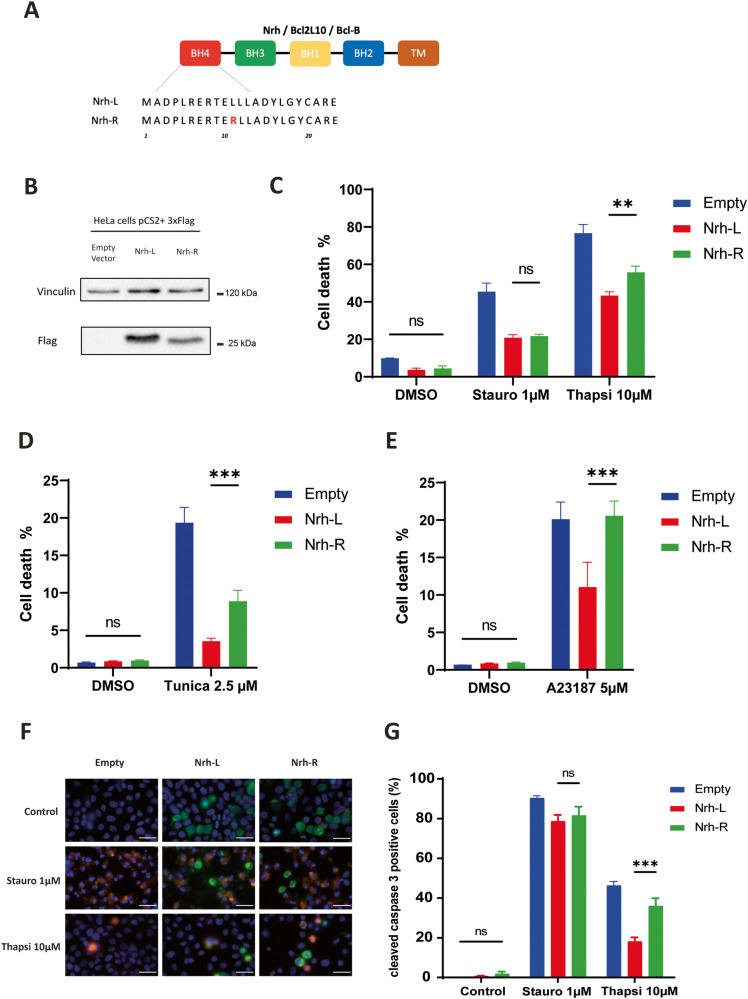
Effect of Nrh-L and Nrh-R with respect to staurosporine and Ca^2+^ stress inducers. **A** SNP Leu11Arg of Nrh. Schematic of the Nrh protein displaying BH and TM domains (colored boxes). Location of the L/R polymorphism, giving rise to Nrh-L and Nrh-R isoforms is shown. **B** Western blotting. Nrh expression in HeLa cells transfected with the pCSII empty vector or pCSII vector harboring Flag-tagged Nrh-R or Nrh-L ORFs (50 µg of cell lysate). Vinculin was used for calibration. **C**–**E** Cell death quantification (SytoxGreen^TM^-positive cells), in Hela cells stably transfected with pCSII empty vector, pCSII Flag Nrh-L or pCSII Flag Nrh-R. Histograms show cell death expressed as mean ± SEM (three independent experiments) Cell death quantification was performed on total population in the following conditions: Control (DMSO), for 48 h, Staurosporine (Stauro), 1 μM for 24 h, Thapsigargin (Thapsi), 10 μM for 72 h, Tunicamycin (Tunica), 2,5 μM for 52 h, A23187 calcium ionophore, 5 μM for 66 h. Corresponding time course analyses are shown in Supplementary Fig. S2. **F** Representative image of cleaved Caspase-3 immunostaining (orange) and Flag-tagged Nrh (green) in cells treated with either staurosporine 1 μM for 24 h or 10 μM of thapsigargin for 48 h. Scale bars: 10 μM. **G** Quantification of active Caspase-3 in Flag-tagged Nrh-positive cells as detected by immunofluorescence in (**D**) as percentage of positivity to cleaved caspase-3 staining (mean ± SEM, three independent experiments). Scale bars, 10 μm. Two-way ANOVA test, **P < 0.01, ***P < 0.001, ns (non-significant) *P* > 0.05.

In previous studies, Nrh was characterized as an anti-apoptotic protein mainly located in the endoplasmic reticulum (ER) [8]. Within this location, Nrh is able to control the release of Ca^2+^_ER_ by interacting with the IP_3_R1 channel. Moreover, the *nrh* gene harbors a polymorphism (SNP # rs223192) affecting the N-terminal region of the Nrh protein such that either a leucine (L) or an arginine (R) is found at position 11, at the level of the BH4 domain (Fig. 1A). Interestingly, the L/R polymorphism is located within the BH4 region at the N-terminus of Nrh, a region that was reported to interact with the ligand binding domain of human IP_3_R1 (hBD) [8]. This prompted us to test whether the L/R polymorphism could affect the anti-apoptotic function of Nrh, and its ability to interact with the IP_3_R1 channel.

**Fig. 2:**
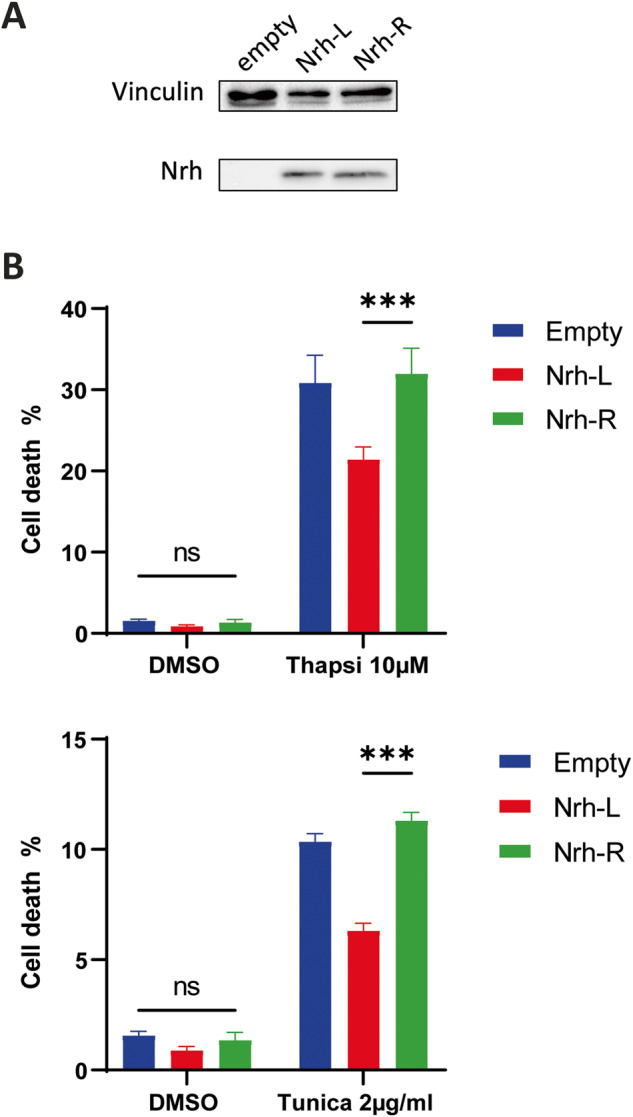
Effect of Nrh-L/R variants on cell death induced by Ca^2+^ stress inducers (MCF-7 breast cancer cells). **A** Western blotting. Nrh expression in MCF-7 cells transfected with the pCSII empty vector or pCSII vector harboring Flag-tagged Nrh-R or Nrh-L ORFs (50 µg of cell lysate). Vinculin was used for calibration. **B** Cell death quantification (SytoxGreen^TM^-positive cells), in MCF-7 cells transfected with pCSII empty vector, pCSII Flag Nrh-L or pCSII Flag Nrh-R. Histograms show cell death expressed as mean ± SEM (three independent experiments) in the following conditions: Thapsigargin (Thapsi) 10 μM for 80 h (top panel) or Tunicamycin (Tunica) 2 μM for 80 h (bottom panel).

Using HeLa cells as a model, we established that ectopic expression of Nrh-L and Nrh-R isoforms inhibited cell death induced by staurosporine, a promoter of the mitochondrial pathway of apoptosis, to the same extent (Fig. 1B, C). Similarly, in these cells, both isoforms protected with equal efficacy against azacytidine, 5-FU and other cytotoxic agents used in chemotherapy (Supplementary Fig. S1). In contrast, the Nrh-L isoform was significantly more effective against Ca^2+^ stress inducers, such as thapsigargin, tunicamycin and the A23817 ionophore (Fig. 1C–E and Supplementary Fig. S2), which was also observed in the MCF-7 breast cancer cell line (Fig. 2 and Supplementary Fig. S3). These observations were corroborated by measuring the activity of Caspase-3, an enzymatic marker of apoptosis (Fig. 1F, G).

### Nrh-L, but not Nrh-R, interacts with the IP3R1 receptor and regulates calcium signaling

**Fig. 3:**
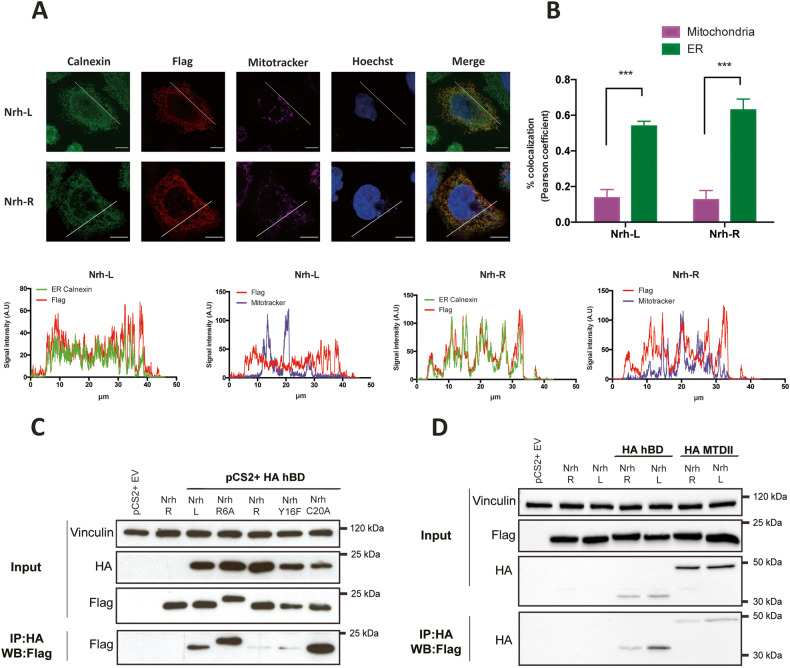
Subcellullar distribution of Nrh-R and Nrh-L variants. **A** Upper left panels: confocal imaging. Immunofluorescence detection of Nrh subcellular localization in HeLa cells expressing Nrh-L and Nrh-R. ER and mitochondria localization was assessed using anti-calnexin antibody and MitoTracker^TM^ Deep Red, respectively (Scale bars: 5 μm). Lower panels: profile plots showing fluorescence intensity along the white segments displayed on merged images. Scale bars 5 μM. **B** Quantification of Nrh colocalization with mitochondrial or ER compartment according to Pearson coefficient. Data are displayed as mean ± SEM (*n* = 3, independent experiments). **C** Co-immunoprecipitations. HeLa cells were co-transfected with vectors expressing IP3R1 binding domain (HA-hBD) and Flag-tagged Nrh (Nrh-L, Nrh-R or Nrh BH4 mutants, as indicated). Immunoprecipitations were performed using anti-HA antibodies, immunoblots were visualized with anti-Flag antibodies. HeLa cells transfected with empty vector (EV) or Flag-tagged Nrh-L alone were used as negative controls. **D** Co-immunoprecipitations. HeLa cells were co-transfected with vectors expressing IP3R1 binding domain (HA-hBD) or IP3R1 transducing domain II (MTDII) with either pCSII empty vector, pCSII Flag Nrh-L or pCSII Flag Nrh-R. Immunoprecipitations were performed using anti-Flag antibodies, immunoblots were visualized with anti-HA antibodies. HeLa cells transfected with empty vector (EV) or Flag-tagged Nrh-L alone were used as negative controls. HeLa cells transfected with empty vector (EV), Flag-tagged Nrh-L or Flag-tagged Nrh-R alone were used as negative controls.

Mechanistically, the location of Nrh at the level of the ER is critical for its effect on Ca^2+^ homeostasis and thapsigargin-induced cell death [8]. Nrh isoforms display a similar subcellular distribution (Fig. 3A, B). In contrast, co-immunoprecipitation (co-IP) experiments displayed in Fig. 3C showed that Nrh-L, but not Nrh-R, co-immunoprecipitated with the hBD region of IP_3_R1, suggesting that the binding affinity of the Nrh-R isoform for IP_3_R1 hBD is significantly lower, compared to Nrh-L. In contrast, co-IP experiments performed with the central region MTDII, indicated that the affinity of Nrh for IP3R1 MTDII is independent of the L/R polymorphism (Fig. 3D).

Overall, the above data showed that the L/R polymorphism affects the Nrh/IP3R1 complex at the level of the hBD region, suggesting consequences regarding IP_3_-dependent Ca^2+^ permeability of the IP3R1 channel.

**Fig. 4:**
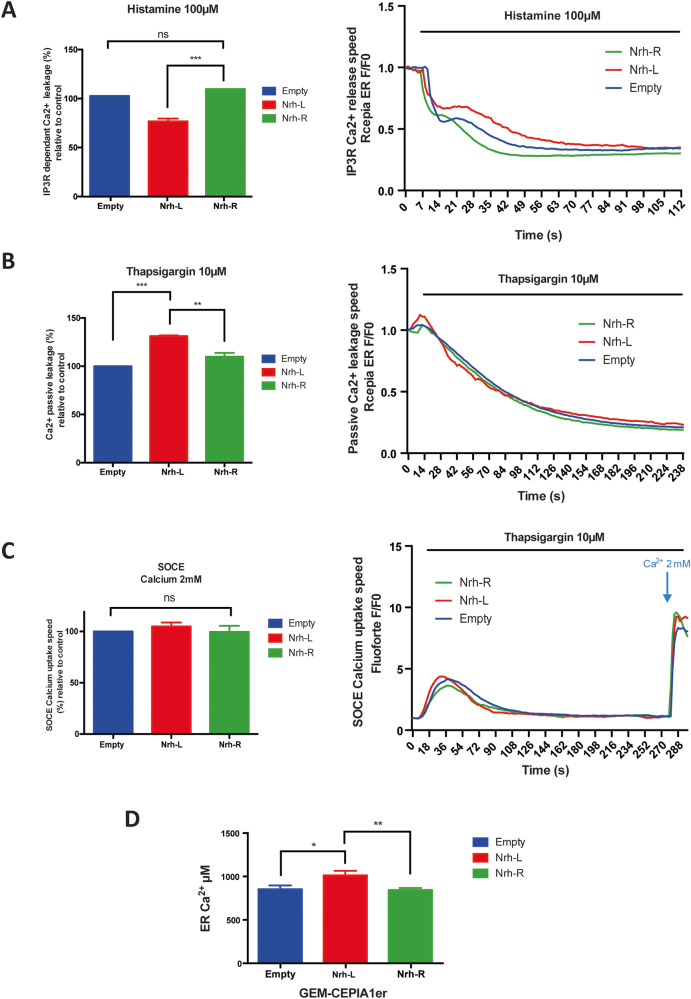
Effect of Nrh-R and Nrh-L isoforms on Ca^2+^ trafficking. **A** Left panel: Quantification of the slope coefficient of Ca^2+^_ER_ release after histamine injection (100 μM) in stable HeLa cells lines (pCSII empty vector, pCSII Flag Nrh-L, pCSII Flag Nrh-R). Prior to the experiment cells were transfected with R-Cepia1er, a Ca^2+^_ER_ specific probe. Right panel: curves from one representative experiment. Slope coefficient was measured for 10 s, starting 10 s after histamine injection. **B** Left panel: Quantification of the slope coefficient of Ca^2+^_ER_ release after thapsigargin injection (10 μM) in stable HeLa cells lines (pCSII empty vector, pCSII Flag Nrh-L, and pCSII Flag Nrh-R). Prior to the experiment cells were transfected with R-Cepia1er. Right panel: curves from one representative experiment. Slope coefficient was measured for 10 s, starting 10 s after histamine injection. **C** Left panel: Quantification of the slope coefficient of SOCE activation induced by Ca^2+^ injection (2 mM) after 4 min of Ca^2+^_ER_ release by thapsigargin (10 μM) in stable HeLa cells lines (pCSII empty vector, pCSII Flag Nrh-L, pCSII Flag Nrh-R). Prior to the experiment, cells were incubated with Fluoforte^TM^ (5 μM) to detect Ca^2+^_Cyt_. Right panel: curves from one representative experiment. Slope coefficient was measured for 10 s, starting 6 s after 2 mM Ca^2+^ injection. **D** Ca^2+^_ER_ pool measurement using GEM-CEPIA1er ratiometric sensor. Nrh-L, but not Nrh-R slightly increases Ca^2+^_ER_ levels, the difference between Nrh-L and Nrh-R is statistically non-significant. All displayed histograms show means ± SEM (*n* = 3, independent experiments). One-way ANOVA test: *p < 0.05, **p < 0.01, ***p < 0.001.

To test this hypothesis, using HeLa cells as a model, we measured the ability of both Nrh isoforms to modulate the release of Ca^2+^_ER_, following treatment with histamine, an activator of phospholipase-C causing rapid increase in IP_3_ levels and subsequent IP_3_R1 channel opening. Indeed, measurements made with the R-Cepia1er fluorochrome showed that the Nrh-R isoform lacked the ability to prevent the release of Ca^2+^_ER_ following histamine treatment (Fig. 4A).

At the ER level, several proteins of the Bcl-2 family have been described as being involved, in addition to the IP_3_-dependent Ca^2+^_ER_ release, in the passive Ca^2+^_ER_ output, which is independent of IP_3_. This so-called "passive" output can be measured by blocking the uptake of Ca^2+^ by the ER with thapsigargin, an inhibitor of the SERCA pump. The results depicted in Fig. 4B show that the passive Ca^2+^_ER_ leakage was effectively increased by Nrh-L, and to a lesser extent by Nrh-R. These observations are in close correlation with their respective capacities to inhibit thapsigargin-induced cell death (see Fig. 1).

However, neither isoform appeared to significantly affect the incoming Ca^2+^ flux from outside the cell, referred to as store-operated Ca^2+^ entry (SOCE, see Fig. 4C).

Collectively, these data show that Nrh-L and -R isoforms act differently on the response to Ca^2+^ stress induced by thapsigargin and histamine. In resting conditions, Nrh-L appeared to slightly increase Ca^2+^_ER_ levels, whereas Nrh-R had no effect (Fig. 4D).

Taken together, the above results show that Nrh-R is less protective with respect to Ca^2+^ stress, providing a mechanistic insight into the functional consequences of the L/R polymorphism, as it appears to affect the ability of Nrh to interact with the IP3R1 Ca^2+^ channel and control Ca^2+^ efflux from the ER and downstream cell death.

### Nrh polymorphism is associated with resistance to therapy

In an attempt to figure out the significance of the L/R polymorphism with regards to clinical outcome, we analyzed the distribution of this polymorphism in a representative set of the general population (Gnomad datset GRCH37/HG19, *n* = 177,982) as well as in the "Signal Phare" breast cancer patient cohort (*n* = 8356). This analysis revealed that 9.7% and 10.99% of individuals harbor the R/R homozygous genotype in the general population and in the patient cohort, respectively (Table 1). Thus, such a limited difference does not indicate that the SNP # rs223192 polymorphism can be predictive of breast cancer susceptibility.

**Table 1:** Distribution of Nrh-L and Nrh-R isoforms (SNP # rs223192) among a representative set of the general population worldwide, according to Gnomad dataset GRCH37/HG19, and of rs2414131 in the breast cancer cohort (Signal Phare, French National Cancer Institute, INCa).

	Total	L/L	L/R	R/R	*P* value
All population (gnomad dataset GRCH37/HG19)	177982 (100%)	103704 (58.3%)	56997 (32.0%)	17281 (9.7%)	<0.0001
Signal Phare (Breast cancer cohort)	8356 (100%)	3610 (43.2%)	3828 (45.81%)	918 (10.99%)	

Number of individuals displayed in boxes (percentage in brackets). Statistical significance shown on the right (*P* value, chi-square test).

On the other hand, growing evidence in the literature indicates that Nrh may play a role in the response to cell death-inducing agents, including cancer drugs [8, 15]. We therefore investigated if the L/R polymorphism could be correlated with chemoresistance. First, using the MCF-7 cell line as a model of breast cancer cells, we noticed that Nrh-L-expressing cells were more resistant than Nrh-R-expressing ones to paclitaxel, a front line chemotherapy drug for breast cancer which was reported to trigger Ca^2+^_ER_ -dependent cell death [20] (Supplementary Fig. S4).

Second, we examined the response to neoadjuvant chemotherapy (Anthracycline/Taxane) in a cohort of patients from the Centre Léon Bérard (CLB). Indeed, as shown in Table 2, homozygous R/R patients responded better to treatment (42.1% complete response to chemotherapy) than L/L or L/R patients, among whom only 20.4% showed complete response to chemotherapy. This polymorphism is not correlated with hormone receptor status, indeed the ER+/ER− ratio is not statistically different among these three subpopulations (namely LL, LR and RR, see Table S1) or with other features such as patient age, tumor size, node status, histologic grade (see supplementary Table S2 for patient information). Thus, these data support that the Nrh L/R polymorphism might be a valuable indicator of response to chemotherapy in the context of breast cancer.

**Table 2:** Response to chemotherapy as a function of Nrh L/R polyporphism.

Bcl2l10 breast/CLB genotype	Test population size	Residual cancer burden (RCB)		Statistical analysis (Fischer’s exact test).	
		PCR	RCB I-III	OR (95%) CI	*P* value)
R/R	19	8 (42.1%)	11 (57.9%)	1.00 *(ref)*	0.044
L/R + L/L	156	32 (20.5%)	124 (79.5%)	0.3548 *(0.1283–0.9708)*	
Total	175	40 (22.9%)	135 (77.1%)		

Residual Cancer Burden (RCB) score was determined through pathologic section of primary breast tumor. PCR (pathologic complete response) was considered to be the best response to chemotherapy, actually corresponding to remission, whereas (RCB I-III) correspond to detrimental outcome. Number of individuals displayed in boxes (percentage in brackets). Statistics are shown on the right. Analyses were performed using Fischer’s exact test with an Odds Ratio (OR) at 95% confidence interval (CI). OR <1 indicated lower odds of association between Nrh-L and pCR. *P* < 0.05 was considered to be statistically significant.

## Discussion

### Nrh polymorphism and chemoresistance

Expression of *bcl2L10/nrh* has been repeatedly reported as a poor prognostic marker, including in acute myeloid leukemia, colorectal cancer and breast cancer. Moreover, Nrh expression in breast cancer was shown to shorten DMFS [8]. Here, we hypothesized that the expression of the Nrh-R isoform may not have the same detrimental outcome as its Nrh-L counterpart. Indeed, breast cancer patient cohort analyses revealed that Nrh-R expression may be predictive of a higher sensitivity to chemotherapy. Moreover, we show in the present study that Nrh-R-expressing cells are more sensitive to Ca^2+^-stress inducers, including thapsigargin, than Nrh-L cells. At the molecular level, such increased sensitivity may be explained by the loss of interaction between Nrh-R and IP3R1 at the hBD level. Consequently, Nrh-R may not control Ca^2+^_ER_ homeostasis as efficiently as Nrh-L, cells thus being unable to deal with ER stress. Mechanistically, the BH4 domain is critical for the interaction with IP3R1 hBD, contributing to it anti-apoptotic activity. However, the L/R polymorphism might not affect interactions aside from that with the IP_3_R1 hBD. Indeed, both Nrh variants interacted with the modulating domain II (MTDII) with similar affinities (Fig. 3D). Moreover, as shown in Supplementary Fig. S1, Nrh-L- and Nrh-R-expressing cells displayed the same level of resistance to azacytidine and etoposide, unlike thapsigargin, indicating that the Nrh-R variant still confers protection against certain death-inducing agents.

### Nrh and calcium-dependent apoptosis

Collectively, our data suggest that Nrh-L confers better protection with regard to Ca^2+^ stress inducers, which may in part underly chemoresistance.

At the level of the ER, Nrh appears to affect both IP_3_-dependent (active) and -independent (passive) Ca^2+^_ER_ release. Indeed, Nrh-L, but not Nrh-R, efficiently decreases active Ca^2+^_ER_ release. However, we observed that Nrh-L fosters passive Ca^2+^_ER_ leakage more effectively, compared to Nrh-R, as measured in presence of thapsigargin. Both effects may contribute to the observed increased chemoresistance in Nrh-L-expressing cells. Indeed, a decrease in Ca^2+^_ER_ was reported to prevent mitochondrial outer membrane permeabilization and subsequent apoptosis [21, 22].

### Nrh-R expression as a prognostic factor

Cohort analyses reported in the present study confirm that Nrh-R expression may be a better prognostic marker regarding response to chemotherapy and further support the relevance of targeting the Nrh/IP3R1 complex for cancer treatment. However, our data do not suggest an impact of the L/R polymorphism regarding breast cancer onset. In this respect, it should be noted that, in contrast, Fabiani and colleagues reported lower frequency of the Nhr-R allele in patients with de novo myelodysplasic syndromes [18], indicating that different conclusions may be drawn, depending on the disease.

Finally, by highlighting the L/R Nrh polymorphism as a predictive factor of tumor progression, our observations support the idea that the ability of cancer cells to hijack the mechanisms controlling Ca^2+^ homeostasis may offer interesting perspectives for targeted therapies.

## Materials and methods

### Drugs and reagents

5-FU (F6627, Sigma), Azacytidine, (A2385, Sigma), Etoposide (E1383, Sigma), Fluoforte (ENZ-2204, ENZO), Gemcitabine (G6423, Sigma), Hoechst 33342 #4082, Cell Signaling), Mitotracker (M22426, Invitrogen), Thapsigargin (BML-PE180, Enzo), Tunicamycin (T7765, Sigma), A23817 (C7522, Sigma), Staurosporine (S4400, Sigma), Histamine (H7125, Sigma).

### Vectors and constructions

For immunoprecipitation assays, Nrh and IP3Rs ORFs, were cloned into the pCS2^+^ vector (Biolabs) between the ClaI and XhoI restriction sites.

To obtain Hela cells stably expressing Nrh-L and Nrh-R, corresponding ORFs were cloned into the pCSII lentiviral vector as described [23].

R-CEPIA1er and GEM-CEPIA1er plasmids were from Addgene (#58216 and #58217, respectively).

### Cohorts and tumor samples

Distribution of NhL/R variants (SNP # rs223192) was analyzed among a representative set of the general population worldwide (Gnomad dataset GRCH37/HG19, 17,7982 individuals) and a prospective clinical cohort referred to as Signal Phare. This latter cohort was specifically designed to study constitutional genetics in patients with breast cancer. This cohort, funded by the French National Cancer Institute (INCa), consists of over 9800 patients diagnosed with breast cancer, recruited through a network of clinicians across France from May 2006 to December 2013. Information on the SNP status of rs2414131, which is strongly associated with the SNP studied in the present work (rs223192), was available in 8356 patients.

Regarding response to chemotherapy, we screened 175 female patients (median age 50 years) with operable primary breast cancer (invasive breast cancer negative for HER2) who underwent neoadjuvant therapy before surgery at the CLB between 2006 and 2015. Axillary lymph node (LN) invasion was assessed by sentinel node and/or level I and II axillary dissection. Tumor size was defined on the tumor specimens at the time of surgery. Tumors were considered ERα- or PR-positive if they displayed a nuclear staining in 10% or more of tumor cells, as detected by immunohistochemistry. Tumors were considered positive for HER2 expression if they had three positive staining by immunohistochemistry or two positive staining by HER2 amplification detected using FISH. The data exported from the patients’ files for analysis are consigned in Supplementary Table S1. Written informed consent was obtained from each patient and the study protocol is reported according to the REMARK criteria and was done according to French regulations and approved by the ethics committee of the Centre Léon Bérard [24].

The SNP status of rs223192 was directly determined on archival formalin-fixed paraffin-embedded tissues by TaqManTM probe (Biosystems Assay probe #4351379). Response to the neoadjuvant chemotherapy (Anthracycline/Taxane) was determined using Residual Burden Score (RCB) on tumor samples after surgery.

### Cell culture

HeLa and MCF-7 cells were obtained from the ATCC and cultured on collagen-coated dishes in DMEM (Gibco, USA) supplemented with 10% fetal bovine serum, streptomycin and penicillin (100 U.mL^−1^). Cell lines were routinely tested for mycoplasma contamination using Mycoalert kit (Lonza) and authenticated by single nucleotide polymorphism profiling (Multiplexion GmbH).

For the generation of Nrh-expressing cell lines, HeLa cells were transfected with pCSII empty vector, pCSII Flag-tagged Nrh-L isoform or Flag-tagged Nrh-R isoform (SNP # rs223192). 24 h after transfection, puromycin (1 µg/ml) was added to the medium to select stably transfected cells.

### Calcium measurements

Before Ca^2+^ imaging, 30,000 cells were plated in Nunc^TM^ Labtek® chambered coverglass. For subcellular ER Ca^2+^ imaging, HeLa cells were transfected with R-CEPIA1er probe, 2 days prior to imaging using X-tremeGENE HP DNA (Roche). Before the experiment, cells were incubated with a Ca^2^ + -free Balanced Salt Solution (BSS) (121 mM NaCl, 5.4 mM KCl, 0.8 mM MgCl_2_, 6 mM NaHCO_3_, 5.5 mM D-glucose, 25 mM HEPES, pH 7.3). After 10 s of measurement, either 100 μM histamine or 10 μM of Thapsigargin in BBS. Six samples were analyzed per experiment. At least three independent experiments were carried out.

For calcium refilling assays, cells were loaded with 5 μM of FluoForte^TM^ Ca^2+^ probe (Enzo) in BBS during 30 min at 37 °C. After injection of 10 µM of thapsigargin, 2 mM of Ca^2+^ was added to the mix.

For Calcium pool measurement, cells were transfected with GEM CEPIA1er, 2 days prior to imaging, and incubated in BBS with 2 mM of Ca^2+^. Time-lapse fluorescence values were collected using a Zeiss LSM 780 confocal microscope. The images were captured at a rate of one frame every 2 s using a ×40 objective. All images were analyzed by ImageJ software.

### Apoptosis assays

HeLa cells were treated either with staurosporine (1 μM for 24 h), thapsigargin (10 μM for 72 h), etoposide (25 μM for 48 h), azacytidine (25 μM for 72 h) and doxorubicin (10 μM for 72 h). For kinetic experiments, 20,000 cells were seeded onto a 96-well plate 12 h prior to treatment. Images were acquired using an Incucyte ZOOM^TM^ every hour from 0 to 72 h at ×4 magnification. Dying cells were determined using Sytox^TM^ Green Nucleic Stain (0.5 μM, ThermoFischer Scientific). Data were processed using a dedicated algorithm. Number of Sytox^TM^ Green positive nuclei was normalized to cell confluence for calibration purposes.

### Immunoprecipitation assays

40,000 HeLa cells were transfected with the indicated vectors and grown for another 48 h in 6-well plates. Cells were lysed in TNE buffer (10 mmol/L Tris-HCl, 200 mmol/L NaCl, 1 mmol/L EDTA (pH 7.4), 1 mmol/L β-glycerophosphate, 1 mmol/L sodium orthovanadate, 0.1 mmol/L sodium pyrophosphate, 0.2% NP-40, protease and phosphatase inhibitor (Roche)). Lysates were precleared with protein G-Sepharose beads (Sigma) for 2 h at 4 °C, then incubated overnight with 2 µg primary antibodies (Flag or HA). Extracts were incubated with protein G-Sepharose beads for 3 h. Immunoprecipitated fractions were washed three times with TNE buffer, and analyzed by immunoblotting. Alternatively, FlagM2 magnetic beads (Sigma) could also be used as a replacement for primary antibodies and protein G-Sepharose.

### Immunoblotting

For kinetic experiments, 40,000 cells were seeded in 6-well plates. Cells were treated prior to harvesting with corresponding drugs. Cell pellets were prepared using RIPA solution then separated via SDS-PAGE and transferred to a nitrocellulose membrane. Membranes were blocked for 1 h in 5% milk or BSA TBS solution followed by overnight incubation with primary antibodies at 4 °C. Membranes were washed for 3 × 15 min at RT in TBS-T (0.1%). Goat anti-mouse/rabbit HRP-conjugated secondary antibodies (Dako, 1:3000) were incubated for 1 h in 5% milk at RT followed by 3 × 15 min at RT in TBS-T. Membranes were exposed to Lumi-Light Western blotting substrate (Roche) and detected on Bio-rad Chemidoc^TM^ imagers.

Primary antibodies used: Vinculin (Santa Cruz #sc-55465; 1:2000), FlagM2 (Sigma #F1804, 1:1000), HA (Sigma #H6908, 1:1000).

### Immunofluorescence

Cells were seeded onto a glass coverslip in 12-well plates at 50% confluence. After 24 h and prior to fixation, cells were incubated with mitochondria-staining dye (MitoTracker^TM^ Red CMXRos, Life Technologies) for 20 min at 37 °C and then fixed with 4% paraformaldehyde. Paraformaldehyde was discarded and cells were washed with PBS. Cells were incubated for 30 min with blocking buffer (0.1% Triton X100, 3% BSA in PBS), incubated for 1 h at RT with primary antibodies and then incubated 1 h with Alexa fluor® secondary antibodies (488, 568 nm, Invitrogen). Hoechst 33342 (1:10,000, Invitrogen #H3570) in TBS-T was added to stain the nuclei of cells. Coverslips were mounted with a Dako mounting medium. Images were acquired using a Zeiss 780 confocal microscope. For apoptosis assay after 1 µM of Staurosporine during 24 h or 10 µM Thapsigargin treatment for 72 h, the same procedure was performed without mitochondria staining dye. Primary antibodies included: FlagM2 (Sigma #F1804, 1:1000), Calnexin (Cell Signaling #2679, 1:1000), cleaved caspase-3 (Cell Signaling #9661S, 1:400).

### Statistical analyses

#### In cellulo assays

All experiments were performed in triplicate. Statistical analyses were performed using Prism (GraphPad). Average value +/− SEM is shown. Two-way ANOVA test was used for statistical analysis to compare the effect of different drugs, one-way ANOVA test is done for weigh the Ca^2+^ trafficking. The tests are two-sided, the normal distribution and the equality of the variance was calculated with Shapiro-Wilk’s test and Bartlett’s test, respectively. A *p*-value equal to or under 0.05 was considered to be statistically significant.

#### Population and cohorts

Within each cohort the analyses were carried out on all individuals within the cohort for whom the required information was available (Gnomad dataset: *n* = 177,982; Signal/Phare: *n* = 8356; Centre Léon Bérard: *n* = 175). To analyze the distribution of populations, a chi-square test was performed. The correlation between expression of Nrh L/R variants and clinicopathologic factors was calculated using Fisher’s exact test. A *p*-value equal to or under 0.05 was considered to be statistically significant.

Statistical significance between two groups was analyzed using the one-tailed Student’s t-test. All statistical tests regarding data analysis were two-sided.

The correlation between Nrh protein expression and clinicopathologic factors was calculated using Fisher’s exact test. A *p*-value equal to or under 0.05 was considered to be statistically significant.

## Supplementary information

Supplementary Figures

Supplementary table S1

Supplementary table S2

Reproducibility checklist

## Data Availability

All data generated or analyzed during this study are included in this published article (and its supplementary information files).
