# Supplementary material for: Nrh L11R single nucleotide polymorphism, a new prediction biomarker in breast cancer, impacts endoplasmic reticulum-dependent Ca2+ traffic and response to neoadjuvant chemotherapy
Source: Cell Death Dis. 2023 Jul 1;14(6):392. doi: 10.1038/s41419-023-05917-7 (PMC10313725; doi:10.1038/s41419-023-05917-7)
Supplement: Supplementary file 1 — Supplementary Figures [file 41419_2023_5917_MOESM1_ESM.docx]

**Supplementary Figure S1. Effect of Nrh-L/R variants on cell death induced by chemotherapy agents.**

**
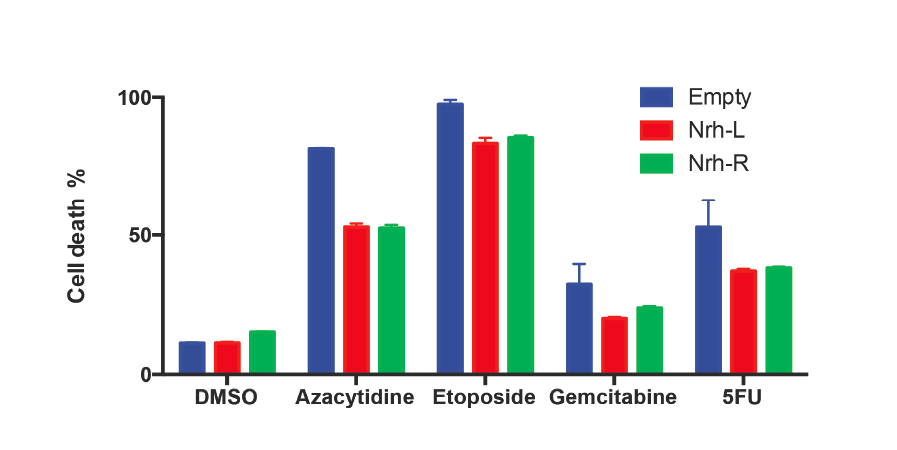
**

HeLa cell were transfected by the following plasmids: (pCS2^+^), (pCS2^+^-Flag Nrh-L) or (pCS2^+^-Flag Nrh-R), were treated with either Azacytidine (50μM, 40 hours), Etoposide (25μM, 72 hours), Gemcitabine (5μM, 72 hours) or 5-Fluorouracil (5FU, 150μM, 72 hours). DMSO-treated cells were used as negative control. Cell death percentage at the corresponding time points is illustrated by histograms.

**Supplementary Figure S2. Effect of Nrh-L and Nrh-R with respect to Ca^2+^ stress inducers (HeLa cells, time course analyses).**

A

B

C

Cell death quantification (SytoxGreen^TM^-positive cells) in Hela cells stably transfected with pCSII empty vector, pCSII Flag Nrh-L or pCSII Flag Nrh-R. Time course analyses were carried out on cells treated as follows, Thapsigargin (TG) 10 μM (A), Tunicamycin 2μM (B), A23187 calcium ionophore 5μM (C). DMSO-treated cells were used as negative controls. Representative results from at least three independent experiments.

**Supplementary Figure S3. Effect of Nrh-L/R variants on cell death induced by Ca^2+^ stress inducers (MCF-7 breast cancer cells, time course analyses).**

**A**

**B**

Cell death quantification (SytoxGreen^TM^-positive cells), in MCF-7 cells transfected with pCSII empty vector, pCSII Flag Nrh-L or pCSII Flag Nrh-R. (A) Cells treated with 10 μM Thapsigargin (Tapsi) or DMSO (negative control). (B) Cells treated with 2μM Tunicamycin (Tunica) or DMSO (negative control). Representative results from a least three independent experiments.

**Supplementary Figure S4. Effect of Nrh-L/R variants on cell death induced by paclitaxel (MCF-7 breast cancer cells).**

Cell death quantification (SytoxGreen^TM^-positive cells), in MCF-7 cells transfected with pCSII empty vector, pCSII Flag Nrh-L or pCSII Flag Nrh-R. Histograms show cell death expressed as mean ± SEM (n = 3 representative experiments) following treatment with Paclitaxel 2μM for 8h (left panel). Corresponding time course analyses are shown (right).
