# Supplementary material for: Nrh L11R single nucleotide polymorphism, a new prediction biomarker in breast cancer, impacts endoplasmic reticulum-dependent Ca2+ traffic and response to neoadjuvant chemotherapy
Source: Cell Death Dis. 2023 Jul 1;14(6):392. doi: 10.1038/s41419-023-05917-7 (PMC10313725; doi:10.1038/s41419-023-05917-7)
Supplement: Supplementary file 2 — Supplementary table S1 [file 41419_2023_5917_MOESM2_ESM.pdf]

Genotype	ER+	ER-	P Value (chi square test)
LL	46 (60%)	31 (40%)	0,5946
LR	44 (59%)	30 (41%)	
RR	9 (47%)	10 (53%)	

Table S1. Distribution of ER+ and ER- patients according to genotype (LL, LR , LR).
Number of individuals are displayed in boxes (percentage in brackets).
Statistical significance is shown on the right (P value, chi square test).
The statistical analysis shows no significant difference in the ER+ / ER- ratio between these three populations. Analyses were performed on the individuals of the Centre Leon Bérard cohort for whom the ER status was available (n=170)
