# Supplementary material for: Nrh L11R single nucleotide polymorphism, a new prediction biomarker in breast cancer, impacts endoplasmic reticulum-dependent Ca2+ traffic and response to neoadjuvant chemotherapy
Source: Cell Death Dis. 2023 Jul 1;14(6):392. doi: 10.1038/s41419-023-05917-7 (PMC10313725; doi:10.1038/s41419-023-05917-7)
Supplement: Supplementary file 3 — Supplementary table S2 [file 41419_2023_5917_MOESM3_ESM.pdf]

Table S2 : Clinical and biological characteristics of patients with invasive breast carcinomas negative for HER2 investigated in this study

ID	L11R status	Age (years)	Tumor size (mm)	SBR grade (before neoadj chemotherapy)	ER status	PR status	Nbr of invaded lymph nodes	RCB	Neoadjuvant chemotherapy A : Antracyclines T : Taxane	Months after diagnosis	Alive : 1 Dead : 2	Remission 1 = yes 2 = no
6	LL	30	2x1	2	pos	pos	1	RCB-I	AT	69	1	2
8	LL	38	40x30	3	neg	neg	1	RCB-II	AT	7	1	1
14	LL	57	2x1	3	pos	pos (weak)	0	RCB-I	AT	8	1	1
18	LL	34	20 x 11	2	pos	pos	0	RCB-II	AT	83	1	1
21	LL	55	70 x 50	3	neg	neg	9	RCB-III	AT	14	2	2
22	LL	60	8x33	3	pos	neg	0	RCB-I	AT	89	1	2
180	LL	57	40x40	2	pos	neg	0	RCB-II	AT	156	1	1
181	LL	50	20x10	2	pos	pos	1	RCB-II	AT	22	1	1
191	LL	55	35x35	2	pos	pos	1	RCB-III	AT	23	1	1
193	LL	63	0	3	neg	neg	0	pCR	AT	133	1	1
194	LL	54	0,2x0,2	3	neg	neg	2	RCB-II	AT	46	1	1
199	LL	42	22x20	3	neg	neg	2	RCB-II	AT	22	2	2
205	LL	48	0	3	neg	neg	0	pCR	AT	132	1	1
206	LL	63	1x1	3	neg	neg	0	pCR	AT	39	1	1
209	LL	31	15x10	2	pos (weak)	pos	1	RCB-II	AT	16	1	1
221	LL	41	100X10(	2	pos	pos	3	RCB-III	AT	16	1	1
224	LL	39	15x10	3	pos	pos	1	RCB-II	AT	23	1	1
225	LL	47	13x11	3	pos	pos	1	RCB-II	AT	142	1	1
230	LL	51	70x70	2	pos	pos	3	RCB-III	AT	123	1	1
231	LL	60	35X40	3	neg	neg	12	RCB-III	AT	25	2	2
250	LL	44	3x3x	3	neg	neg	0	RCB-I	AT	117	1	1
258	LL	40	0	2	pos	neg	2	RCB-I	AT	116	1	1
274	LL	43	15X15	2	pos	pos	15	RCB-III	T	120	2	2
275	LL	48	0	3	neg	neg	0	pCR	AT	129	1	1
282	LL	52	5X2	3	neg	neg	0	RCB-I	AT	112	1	1
288	LL	58	55X50	2	pos	pos	6	RCB-III	AT	8	1	1
297	LL	48	50x50	2	NC	NC	8	RCB-III	AT	10	1	1

298	LL	44	30 x 30	3	neg	neg	1	pCR	AT	7	1	1
312	LL	40	0	3	pos	pos	4	RCB-II	AT	7	1	1
315	LL	66	15 x 15	2	pos	pos	1	RCB-II	AT	7	1	1
319	LL	53	15 x 20	2	pos	pos	0	RCB-II	AT	9	1	1
323	LL	62	0	3	neg	neg	0	pCR	AT	106	1	1
325	LL	56	16 x 10	2	pos	pos	4	RCB-III	AT	8	1	1
327	LL	43	0	3	neg	pos	0	pCR	AT	6	1	1
330	LL	51	40 x 40	3	pos (weak)	pos (weak)	1	RCB-III	AT	8	1	1
332	LL	67	80 x 70	3	pos (weak)	pos (weak)	6	RCB-III	AT	14	2	2
333	LL	42	45 x 40	3	neg	pos (weak)	0	RCB-II	AT	91	1	1
338	LL	46	0	3	pos	pos	0	pCR	AT	61	1	1
339	LL	64	0	3	neg	neg	0	pCR	AT	25	1	1
357	LL	62	30x20	2	pos	pos	1	RCB-III	AT	8	1	1
358	LL	37	30x25	3	pos	pos	3	RCB-III	AT	40	1	1
359	LL	55	20x20	3	pos	neg	4	RCB-III	AT	7	1	1
382	LL	55	0	3	neg	pos (15%)	0	pCR	AT	10	1	1
394	LL	50	35x15	2	pos	pos	5	RCB-III	AT	32	1	1
397	LL	40	0	3	pos (weak)	neg	0	pCR	AT	42	1	1
400	LL	56	8x2	3	neg	neg	1	RCB-I	AT	50	1	2
412	LL	74	30x30	2	pos	pos	8	RCB-III	AT	10	1	1
421	LL	62	100x10[illegible]	2	pos	pos	6	RCB-III	AT	29	1	1
426	LL	36	22x10	2	pos	pos	0	RCB-II	AT	51	1	1
427	LL	50	45x30	2	pos	pos	5	RCB-II	AT	38	1	1
428	LL	66	30x30	2	pos	pos	4	RCB-III	AT	6	1	1
430	LL	41	0	2	pos	pos (weak)	1	RCB-I	AT			
435	LL	41	10X3	2	pos	pos	1	RCB-II	AT	13	1	1
442	LL	44	45x30	2	pos	pos	2	RCB-III	AT	18	1	1
443	LL	27	0	3	neg	neg	0	pCR	AT	57	1	1
446	LL	59	30x20	2	pos	pos (weak)	4	RCB-III	AT	8	1	1
452	LL	38	1x1	3	neg	neg	0	RCB-I	AT	67	1	1
455	LL	55	40x20	3	pos	pos	2	RCB-III	AT	48	1	2
457	LL	51	30x25	3	neg	neg	2	RCB-III	AT	31	1	1
458	LL	40	0	3	neg	neg	0	pCR	AT	8	1	1
459	LL	34	60x40	3	pos	pos	1	RCB-III	AT	23	1	2

460	LL	57	0	3	neg	neg	0	pCR	AT	43	1	1
468	LL	60	10 x 5	2	pos	pos	0	RCB-II	AT	31	1	1
470	LL	52	33 x 30	3	pos (weak)	neg	0	RCB-II	AT	11	1	2
474	LL	30	0	3	neg	neg	0	pCR	AT	66	1	1
477	LL	42	6x3	3	neg	neg	0	RCB-II	AT	7	1	1
478	LL	50	6x6	3	pos	pos	2	RCB-II	AT	7	1	1
481	LL	57	7 x 3	3	neg	neg	0	RCB-I	AT	6	1	1
484	LL	65	30x20	2	pos	pos	1	RCB-II	AT	11	1	1
487	LL	43	0	3	neg	neg	0	pCR	AT	69	1	1
488	LL	63	45x45	3	neg	neg	1	RCB-III	T	75	1	1
491	LL	59	9 x 20m	3	pos	neg	0	RCB-II	T	4	1	1
513	LL	69	0	3	neg	neg	0	pCR	AT	20	2	2
516	LL	50	90x60	2	pos	pos	5	RCB-III	AT	11	1	1
541	LL	36	4x3	3	neg	neg	1	RCB-II	AT	19	2	2
552	LL	34	15 x 10	3	pos	neg	0	RCB-II	AT	58	1	1
558	LL	67	15x10	3	neg	neg	4	RCB-II	AT	9	1	1
561	LL	36	10x10	2	pos	pos	4	RCB-II	AT	13	1	1
2	LR	29	55 x 50	3	neg	neg	1	RCB-III	AT	5	1	1
5	LR	45	35X25	3	NC	NC	14	RCB-III	AT	11	2	2
9	LR	57	5 x 2	3	neg	neg	0	RCB-I	T	5	1	1
10	LR	62	25 x 18	2	neg	neg	1	RCB-III	AT	6	1	1
11	LR	67	1 x 1	3	pos	neg	1	RCB-II	AT	68	2	2
17	LR	69	35 x 20	3	pos	pos	0	RCB-II	AT	5	1	1
26	LR	51	70X60	3	neg	neg	11	RCB-III	AT	35	2	2
185	LR	41	0	1	pos	pos	1	RCB-I	AT	133	1	1
197	LR	48	28x28	3	neg	neg	0	RCB-II	AT	137	1	1
200	LR	34	14x14	2	pos	pos	0	RCB-II	AT	109	1	1
207	LR	44	25x20	2	pos	pos	1	RCB-II	AT	134	1	1
210	LR	67	6x5	3	neg	neg	3	RCB-II	AT	41	1	1
216	LR	43	0	3	neg	neg	0	pCR	AT	18	1	1
220	LR	45	20x10	2	pos	pos	2	RCB-III	AT	21	1	1
243	LR	32	15x5	2	pos	pos	2	RCB-II	AT	23	1	1
247	LR	46	28X18	3	pos	pos	5	RCB-II	AT	24	1	1
248	LR	47	0	3	neg	neg	1	RCB-I	AT	75	1	2

254	LR	44	110x110	3	neg	neg	13	RCB-III	AT	14	2	2
264	LR	55	6X6	3	neg	neg	0	RCB-I	AT	92	2	
266	LR	46	0	3	neg	neg	0	pCR	AT	161	1	1
272	LR	38	25x25	1	pos	pos	0	RCB-II	T	115	1	1
277	LR	60	15X10	3	pos	pos	0	RCB-II	AT	113	1	1
278	LR	52	50X45	3	neg	neg	2	RCB-III	AT	26	2	2
279	LR	48	0	3	neg	neg	0	pCR	AT	112	1	1
295	LR	48	0	3	neg	neg	0	pCR	AT	7	1	1
299	LR	56	20 x 10	2	pos	pos	1	RCB-II	AT	8	1	1
301	LR	64	10 x 5	2	neg	neg	5	RCB-II	AT	8	1	1
304	LR	47	18 x 7	2	pos	pos	0	RCB-II	AT	10	1	1
309	LR	43	20 x 18	2	pos	pos	1	RCB-II	AT	8	1	1
314	LR	52	25 x 10	2	pos	neg	4	RCB-II	AT	11	1	1
318	LR	46	10 x 17	2	NC	NC	0	RCB-II	AT	9	1	1
328	LR	49	0	3	pos (weak)	pos	1	RCB-I	AT	56	1	2
343	LR	67	20x10	2	pos	pos	0	RCB-II	AT	7	1	1
344	LR	45	20x20	3	pos	pos	0	RCB-I	AT	34	1	1
345	LR	47	2 x 1	1	pos	pos	0	RCB-I	AT	125	1	1
353	LR	55	10x8	3	pos	pos	0	RCB-II	AT	7	1	1
360	LR	62	25 x 20	2	pos	pos	0	RCB-II	AT	39	1	1
362	LR	45	3 x 1	3	pos (weak)	neg	0	RCB-I	AT	7	1	1
363	LR	31	10x10	2	pos	pos	1	RCB-II	AT	32	1	1
369	LR	57	0	3	neg	neg	0	pCR	AT	79	1	1
374	LR	46	10x7	2	pos	pos	0	RCB-II	AT	9	1	1
375	LR	45	22x20	2	pos	pos	0	RCB-II	AT	41	1	1
379	LR	47	18x12	3	pos	pos	14	RCB-II	AT	23	1	1
380	LR	41	15 x 12	2	NC	NC	0	RCB-II	AT	9	1	1
388	LR	65	50x50	2	pos	pos	3	RCB-III	AT	17	1	1
393	LR	55	20 x 10	3	neg	neg	0	RCB-II	AT	33	2	2
398	LR	54	25x15	3	pos	pos	10	RCB-III	AT	12	1	2
403	LR	30	30 x 30	3	neg	pos (weak)	0	RCB-II	AT	72	1	1
405	LR	40	0	3	pos	pos	0	pCR	AT	7	1	1
409	LR	63	30x25	2	pos	pos	3	RCB-II	AT	14	1	1
411	LR	72	150x100	2	pos	pos	2	RCB-II		75	1	2

413	LR	57	60 x 35	3	neg	neg	0	RCB-II	AT	78	1	1
419	LR	70	35x20	2	NC	NC	10	RCB-III	AT	7	1	1
429	LR	54	45x30	3	neg	neg	5	RCB-III	AT	64	1	1
437	LR	74	0	2	neg	neg	0	pCR	T	28	1	2
451	LR	53	0	3	pos	pos	0	pCR	AT	9	1	1
453	LR	41	0	3	neg	neg	0	pCR	AT	42	1	1
454	LR	44	80x40	2	pos	pos	8	RCB-III	AT	52	1	1
465	LR	36	6x7	2	pos	pos	0	pCR	AT	34	1	1
472	LR	32	0	2	neg	neg	1	RCB-I	AT	8	1	1
483	LR	56	1x1	3	pos	pos	0	RCB-I	AT	7	1	1
495	LR	45	12 x 7	2	pos	pos	0	RCB-II	AT	10	1	1
508	LR	69	20x15	2	neg	neg	1	RCB-II	AT	40	1	1
509	LR	52	20 x 15	3	neg	neg	0	RCB-II	AT	57	1	1
510	LR	75	0	3	neg	neg	0	pCR	T	6	1	1
523	LR	38	0	3	neg	neg	0	pCR	AT	29	1	1
525	LR	51	0	2	pos	pos (weak)	0	pCR	AT	7	1	1
528	LR	40	0	3	pos	pos	1	RCB-I	AT	22	1	2
536	LR	63	25X23	2	pos	pos	1	RCB-III	A	14	1	1
540	LR	53	40x40	2	pos	pos	1	RCB-II	T	9	1	1
543	LR	47	14x18	3	pos	pos	0	RCB-II	AT	24	1	1
554	LR	45	21 x 14	3	neg	neg	0	RCB-II	T	10	1	1
555	LR	60	0	3	neg	neg	0	pCR	AT	10	1	1
560	LR	64	0	3	neg	neg	0	pCR	AT	55	1	1
564	LR	62	5x5	3	pos (weak)	pos (weak)	3	RCB-III	AT	8	2	2
565	LR	44	25x15	2	pos	pos	1	RCB-II	AT	50	1	1
568	LR	58	10x10	3	pos	neg	0	RCB-I	AT	28	2	2
569	LR	49	20x10	2	pos	pos	1	RCB-II	AT	20	1	1
13	RR	40	35 x30	2	pos	pos	1	RCB-III	AT	5	1	1
263	RR	54	13x7	1	pos	pos	0	RCB-II		122	1	1
268	RR	48	37X35	3	neg	neg	1	RCB-III	AT	16	1	2
269	RR	37	40x40	3	neg	neg	0	RCB-II	AT	128	1	1
313	RR	49	30 x 20	2	pos	neg	2	RCB-III	AT	8	1	1
383	RR	36	0	3	neg	neg	0	pCR	AT	99	1	1
402	RR	35	1x1	3	neg	neg	0	pCR	AT	30	1	1

407	RR	55	40x40	3	neg	neg	1	RCB-III	AT	35	2	2
415	RR	56	50x35	2	pos	pos	24	RCB-III	AT	52	2	2
444	RR	54	60 x 60	2	neg	neg	0	pCR	AT	37	1	1
463	RR	60	0	3	neg	pos (weak)	0	pCR	AT	7	1	1
475	RR	42	10 x 7	2	pos (weak)	neg	0	pCR	AT	10	1	1
490	RR	31	0	3	neg	neg	0	pCR	AT	8	1	1
492	RR	38	0	3	neg	neg	0	pCR	AT	15	1	1
497	RR	49	0	3	pos	neg	0	pCR	AT	8	1	1
499	RR	32	20x10	3	pos	pos (weak)	4	RCB-II	AT	7	1	1
522	RR	68	55x40	3	pos	pos (weak)	1	RCB-III	AT	9	1	1
526	RR	54	25X15	2	pos	pos	2	RCB-III	AT	55	1	1
566	RR	43	12 x 12	3	neg	neg	0	RCB-II	AT	40	1	1
